# Treatment-Refractory Scleredema Diabeticorum Managed With Re-irradiation Using Volumetric Modulated Arc Therapy

**DOI:** 10.7759/cureus.69279

**Published:** 2024-09-12

**Authors:** Jayden R Gracie, Ryan Whitaker

**Affiliations:** 1 Radiation Oncology, Vanderbilt University Medical Center, Nashville, USA

**Keywords:** chronic diabetic complication, diabetic skin, radiotherapy in benign disease, scleredema, scleredema diabeticorum, type 1 diabetes mellitus (t1dm), type 2 diabetes mellitus (t2dm), volumetric‐modulated arc therapy (vmat)

## Abstract

Scleredema diabeticorum is a rare connective tissue complication of diabetes, most typically observed in adults with longstanding type 2 diabetes. Patients develop reticular dermis thickening with a peau d'orange appearance at the upper back and neck that occasionally extends over the deltoids and lower back. While considered a benign disorder, it may signify more significant diabetes and can be functionally and cosmetically impairing with insidious onset, few to no effective treatments, and low spontaneous remission potential. Treatment options are limited; however, radiation therapy has shown clinical benefit in severe cases. Common treatment utilizes 20-24 Gy in 10-12 fractions with retreatment sometimes required for adequate symptom control. Here, we present a case of extensive, treatment-refractory diabetic scleredema status post six separate prior treatments with electron radiotherapy with clinical progression of disease, including involvement of anterior neck limiting jaw and neck range of motion. Volumetric modulated arc therapy (VMAT) was utilized to cover all areas of extensive disease and to treat deeper tissues, which we postulated may decrease skin and underlying tissue tightness and provide clinically meaningful improvements in range of motion. A 57-year-old female with longstanding type 1 diabetes and a six-year history of biopsy-confirmed scleredema presented with treatment-refractory disease in 2022 of the upper back, bilateral upper arms, and neck. Her disease distribution included 270 degrees around her neck, making electron therapy unviable. She had previously undergone multiple treatments, including six separate electron radiotherapy treatment courses to her back, shoulders, and posterior neck between 2016 and 2020 (ranging from 12 Gy in 6 fractions to 24 Gy in 12 fractions for each course), psoralen UV-A therapy, physiotherapy, methotrexate, and percussive therapy. To address the disease extending anteriorly around her neck and below her jaw, as well as the posterior involvement of her shoulders, back, and neck, a more extensive VMAT re-irradiation plan was developed. The plan successfully delivered 20 Gy in 10 fractions to areas of clinically evident disease. Toxicity was evaluated using the Common Terminology Criteria for Adverse Events (CTCAE) scoring system at the time of treatment and at the three-month follow-up. Functional improvement, patient satisfaction, skin texture, and induration were assessed during treatment and at her follow-up visit. Following VMAT re-irradiation, the patient denied scorable toxicity and noted the return of mobility in her neck and jaw. We concluded that VMAT-based re-irradiation is a safe and effective option for patients with treatment-refractory scleredema diabeticorum that is non-responsive or not amenable to electron therapy.

## Introduction

Epidemiology and diagnosis

Scleredema adultorum was a term originally coined by Buschke in 1902 referring to non-pitting edematous skin thickening; however, the condition can also be identified by its poorly demarcated borders, hardening of the skin with a leathery texture, erythema or increased skin pigmentation, and decreased sensation over-involved regions [1-3]. Scleredema is caused by collagen fiber hyperplasia, as well as mucopolysaccharide buildup in the deep reticular dermis [2]. In 1968, Graff identified three types of scleredema with scleredema type 1 corresponding to what is now known as Buschke’s scleredema, which follows respiratory infections and scleredema type 2 characterized by a course lasting months to years and is often linked to the development of multiclonal gammopathies and scleredema type 3, now labeled as scleredema diabeticorum [1,4]. Among these types, scleredema type 3 is responsible for half of the cases of scleredema [5].

The incidence of scleredema diabeticorum ranges from 2.5% in outpatient diabetic populations to up to 14% in hospitalized diabetic populations and is more commonly observed in males [2,6]. Most cases occur in insulin-dependent diabetics, and no cases have been described in children [2]. A majority of patients with diabetic scleredema have other co-occurring diabetic complications, including neuropathy and cardiovascular problems [1].

Scleredema is often associated with functional musculoskeletal impairment. In cases with deltoid involvement, the skin tightening and texture changes of scleredema can impair the range of motion (ROM) in the upper extremities, and extensive disease may even cause restrictive lung disease and increase the risk of developing sleep apnea [2]. While the full-thickness excisional biopsy is required to determine the increased dermal thickness diagnostic of scleredema, ultrasound and CT can often provide an approximation [2,7].

Potential treatments

While scleredema often responds poorly to treatment, small case series and case reports have described several therapies to manage the functional impairment caused by the condition.

Glycemic Control

Tight glycemic control is associated with a decreased risk of development of scleredema and other diabetic complications; however, once skin lesions arise tight glycemic control may have limited effectiveness for resolving established lesions [3]. Small series have demonstrated varying degrees of benefit from tight glycemic control on established lesions. In a cohort of 10 patients examined, only half were able to achieve appropriate glycemic control with oral diabetic medications and insulin [5]. While none of the patients without good glycemic control had skin thickness improvement, the five patients with improved glycemic control showed a partial reduction in induration [5]. In another series of four patients with type 1 diabetes (T1DM), a decrease of HbA1c from 9.3 ± 0.7% at baseline to 7.9% ± 1.3% using an implantable insulin pump produced a noticeable decrease in nonpitting edema and redness [8]. Finally, as part of a case series of 30 patients with scleredema diabeticorum treated with various modalities, one patient with dietary modifications alone was completely healed of scleredema [6].

Antibiotics

Antibiotics may be helpful in cases of documented infections with Buschke’s scleredema but have a limited role in scleredema diabeticorum [3]. Only one case of partial response following high-dose penicillin has been reported in diabetic scleredema, involving a patient with T1DM and a 15-year history of scleredema [9].

Phototherapy

Two patients with scleredema achieved complete resolution following 30 and 56 treatments of low-dose UV-A1 phototherapy [10]. A series involving six patients who received low- or medium-dose UV-A1 phototherapy also showed positive results outside of one patient who had to discontinue treatment due to a skin reaction consistent with polymorphous light eruption [11]. Four of the five remaining patients demonstrated moderate to full improvement, with only one patient experiencing relapse 15 months after finishing therapy. In a third study, the combination of UVA-1 phototherapy with physiotherapy and topical corticosteroids provided partial treatment responses [12].

Photochemotherapy (PUVA), which combines the application of psoralen with phototherapy, also may benefit patients with scleredema either as a standalone treatment or in conjunction with other therapies [3,6,13]. In a case report, a 53-year-old male with a 20-year history of type 2 diabetes (T2DM) and painless ill-defined scleredema of the upper back limiting shoulder mobility, PUVA treatment and physiotherapy improved skin elasticity and softness and allowed some return of shoulder ROM [3]. Patients have also demonstrated partial responses to PUVA combined with immunosuppressive agents such as corticosteroids and cyclosporine [6]. Additionally, bath-PUVA therapy, administered over a median of 59 sessions, yielded positive results in a small patient cohort [13].

Colchicine

While zero of two patients treated with colchicine alone per one report experienced improvement, two case reports observed that the addition of colchicine to PUVA therapy led to marked improvement, suggesting synergistic effects [6,14,15]. In one of these reports, a dramatic partial response - in which the patient was finally able to lie supine at night without analgesics - was seen using local PUVA treatment two times per week for 50 total treatments combined with colchicine [14]. Similar markedly improved results were appreciated in a separate case report in response to low-dose broad-band phototherapy plus colchicine treatments [15].

Corticosteroids

Corticosteroids alone completely resolved diabetic scleredema in one patient [6]. In comparison, as part of the same study, physiotherapy alone led to partial responses in three of three patients, and the immunosuppressant cyclosporine alone resulted in incomplete improvement in one patient.

Methotrexate

In the case of extensive scleredema, low-dose methotrexate produced improvement in the induration of a couple of the affected areas after two months; however, after four additional months of treatment, there was no additional improvement [16]. Similarly, five patients on a three-month regimen of low-dose methotrexate showed partial therapeutic response [17].

Radiation 

Although there is limited existing literature on radiation for scleredema diabeticorum, the results of small case studies and series have been promising. For instance, partial improvement was seen in three patients treated with localized electron therapy using doses of 20-24 Gy over 10-12 fractions [18]. In another case, subjective improvement, in the absence of significant change in skin induration, was observed after 20 Gy in 10 fractions with 9 MeV electrons using a single posterior portal [19]. However, retreatment with the same dose six months later alleviated the patient's pruritus, reduced skin induration, and significantly boosted ROM in the neck and bilateral arms. In a case report involving a patient with scleredema secondary to T1DM, 20 Gy in 10 fractions of twice-weekly electrons localized to the affected regions of the neck and torso caused total resolution of scleredema skin findings by three-month follow-up and no adverse reactions [20]. Pulmonary function testing revealed improvement at six months, and the patient remained free of disease at a two-year follow-up.

In three advanced cases of scleredema diabeticorum resistant to prior therapies, Bowen et al. treated the primary site of restrictive skin findings using either an electron beam or a combination of electron beam and photon irradiation, delivering doses between 18 and 21.6 Gy in 1.8-2.0 Gy fractions [7]. Objective improvement in shoulder abduction was documented in each patient, with effects lasting from one to two and a half years. In some of the cases, 9-16 MeV electrons and superflab bolus were used to ensure that 100% of the dose reached the skin surface to the maximum lesion thickness (as defined on CT imaging). All three patients required additional treatments. One patient received a second course of electron-beam radiation to the primary area of restriction one year after the initial treatment as well as radiation to the lower torso. Another patient underwent further radiation to the buttocks and abdomen, while the final patient received additional forearm treatments.

## Case presentation

Our patient is a 57-year-old woman with T1DM managed with an insulin pump. She has a six-year history of scleredema diabeticorum, with non-pitting skin thickening and tightening involving her upper back, bilateral upper arms, and neck. Her T1DM is also complicated by adhesive capsulitis, diagnosed seven years ago, for which she has undergone several shoulder surgeries targeting her rotator cuff, as well as subacromial injections.

Her diagnosis of scleredema was confirmed six years ago via biopsy at an outside institution, and she initially was started on PUVA treatment for approximately three months without results before being referred to radiation oncology at our institution.

Between 2016 and 2020, this patient had undergone electron radiation treatments up to two times a year for relapsed disease. Each treatment provided temporarily improved skin texture as well as arm and shoulder mobility. Her initial radiation course in May 2016 involved 24 Gy in 12 fractions to the upper back and posterior neck. Subsequently, in October 2016, she completed her second course of 24 Gy in 12 fractions targeting the bilateral shoulders. Additional treatments included 24 Gy in 12 fractions to her back in April 2017 with no toxicity, followed by a similar dose in December 2017, with only Common Terminology Criteria for Adverse Events (CTCAE) grade 1 dermatitis and skin itching reported on her shoulders.

In January 2019, she received 24 Gy in 12 fractions over her lower back, neck, and shoulders, with side effects of grade 1 dermatitis and itching. Finally, her most recent electron radiation occurred in March 2020, administered in a protracted course of 12 Gy in 6 fractions to her bilateral shoulders and upper back. This approach aimed to mitigate the risk of long-term side effects from multiple radiation treatments, and she encountered no scorable acute toxicities while achieving a very short-lived complete response.

The patient’s scleredema relapsed, leading to the initiation of low-dose methotrexate therapy in April 2020. Unfortunately, she developed general malaise and had elevated liver enzymes despite no noticeable improvement in her scleredema, prompting treatment cessation in August 2021. She also experimented with percussive devices around that same period and attempted physical therapy and dry needling, but these interventions yielded no appreciable benefits.

In September 2022, due to new and debilitating symptoms hindering her ability to continue her career, she returned to the radiation oncology clinic. Over the course of several months, she had developed severely diminished ROM in her bilateral shoulders, neck stiffness and tightening, and a new limitation in jaw movement due to the anterior extent of her neck disease. On examination, the skin over her posterior neck and upper back exhibited a thickened, woody texture, and her neck had reduced ROM. Grade 1 telangiectasias were noted in previously irradiated areas on her upper back. However, given this anterior distribution of involvement and lack of a flat treatment surface, electron therapy was no longer a viable option.

## Discussion

**Figure 1:**
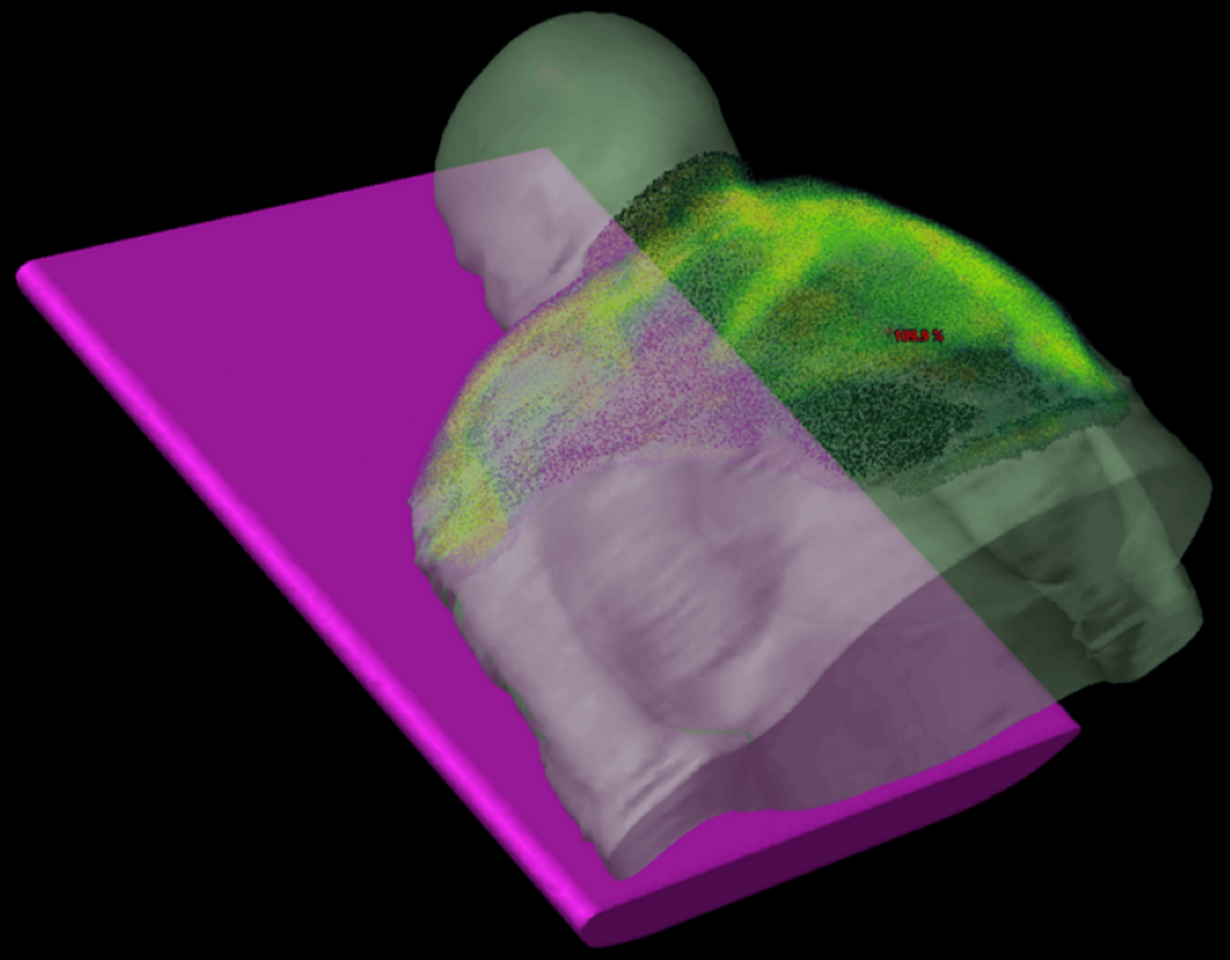
Three-dimensional reconstruction of the VMAT plan This figure demonstrates the dose distribution over the shoulders, middle and upper back, and posterior and lateral neck where scleredema changes were noted. The purple block represents the treatment table.

**Figure 2:**
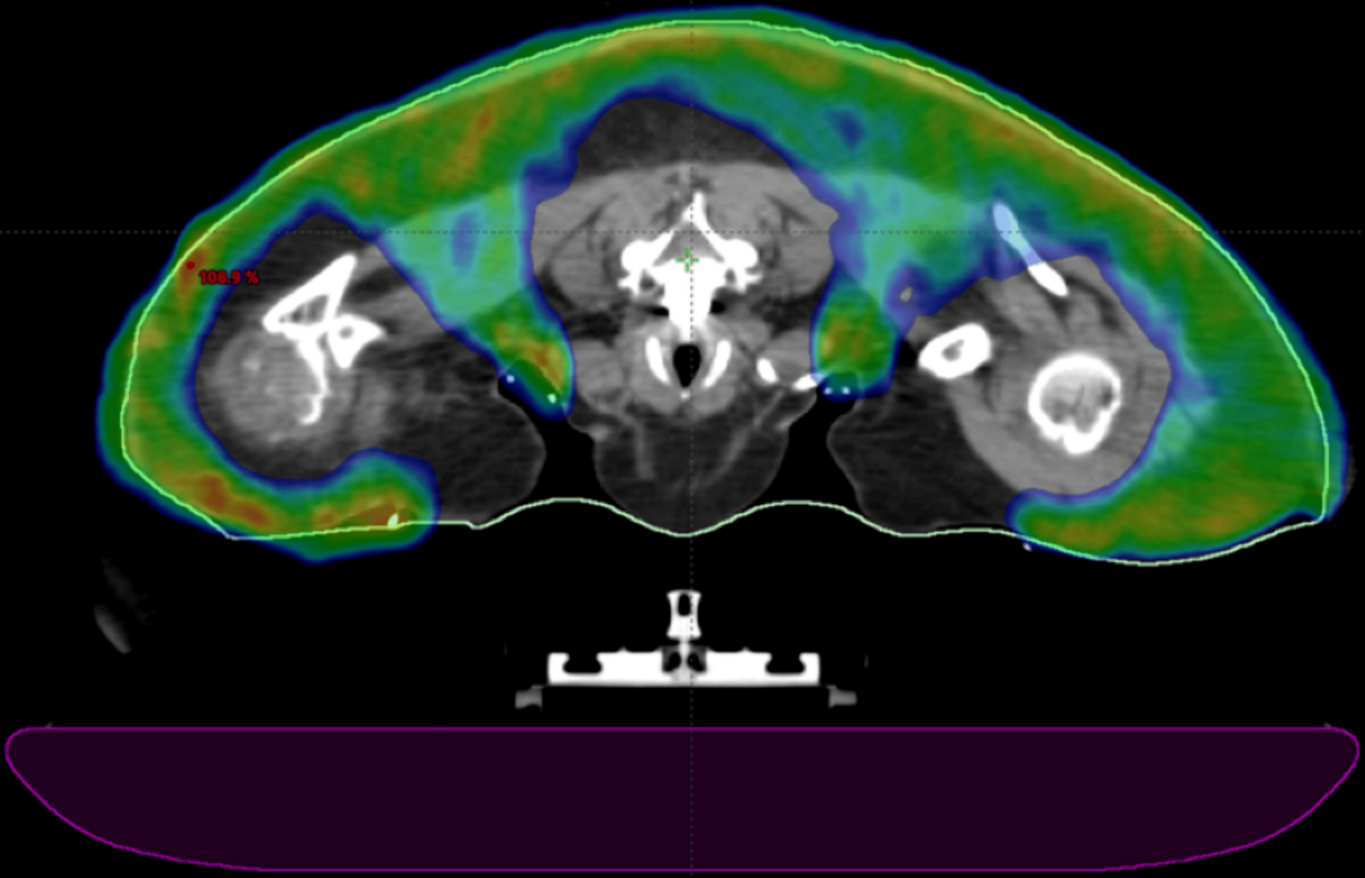
Axial view of the VMAT plan This figure depicts the anterior extent of the treatment coverage around the patient’s neck and shoulders. The treatment table is again represented below the patient in purple.

**Figure 3:**
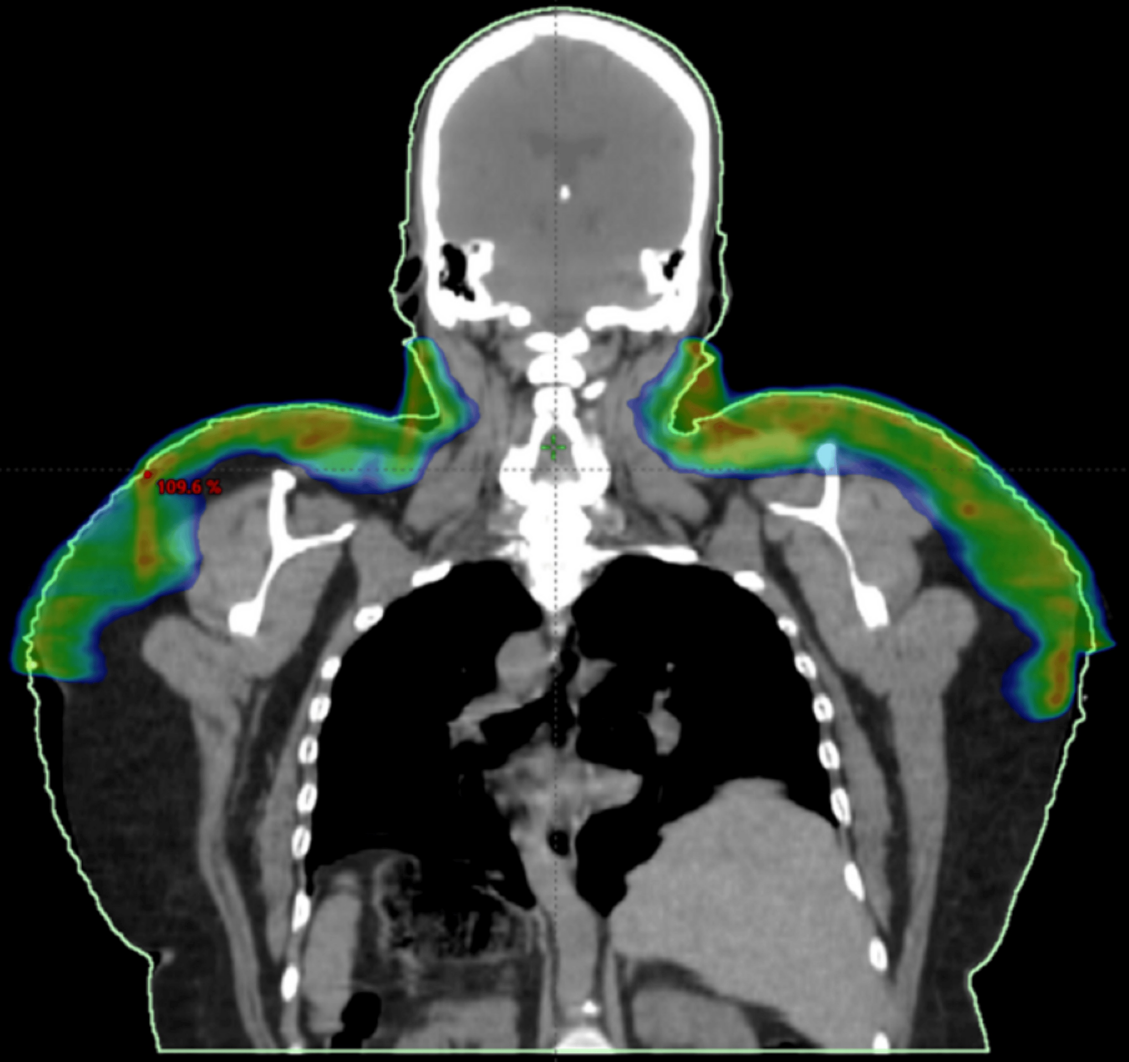
Coronal view of the VMAT plan This figure depicts how the radiation coverage included the entire craniocaudal extent of the patient's lateral neck. The tightness of the skin there had restricted her jaw movements, necessitating treatment coverage to just below the level of the mandible.

**Figure 4:**
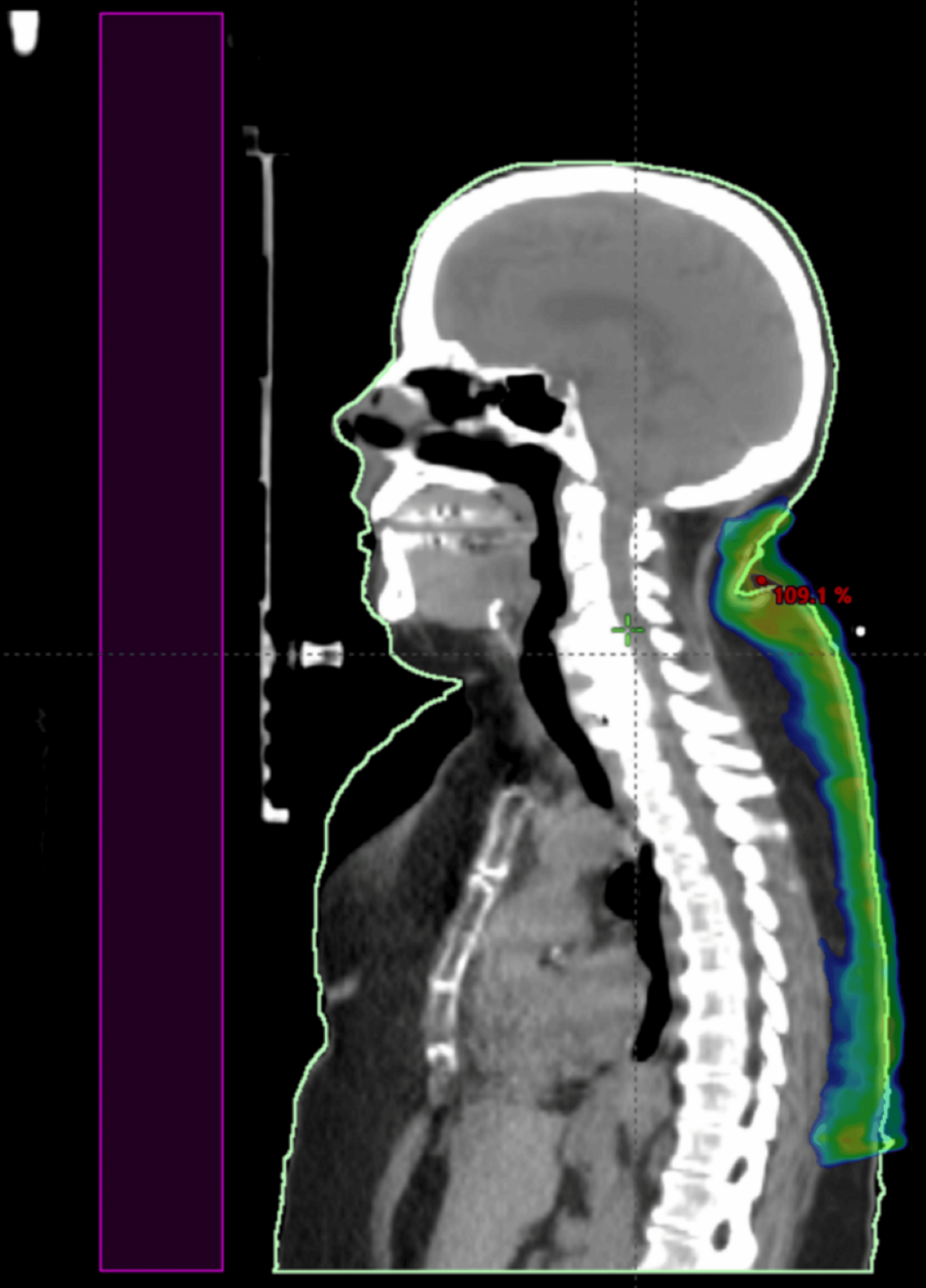
Sagittal view of the VMAT plan This figure demonstrates the craniocaudal extent of treatment coverage from the occiput down to the mid-back.

**Figure 5:**
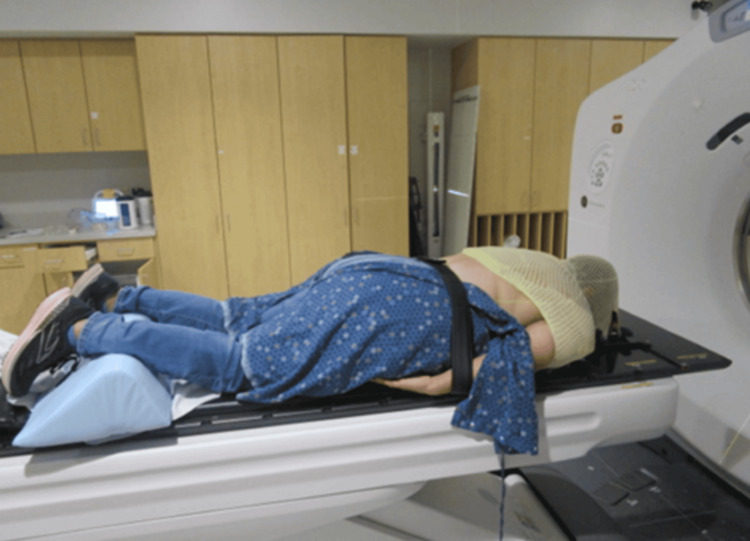
Treatment setup on table This figure shows the patient's prone positioning. A long mask extends over the shoulders and back of the head, and the patient's arms are secured at her sides.

**Figure 6:**
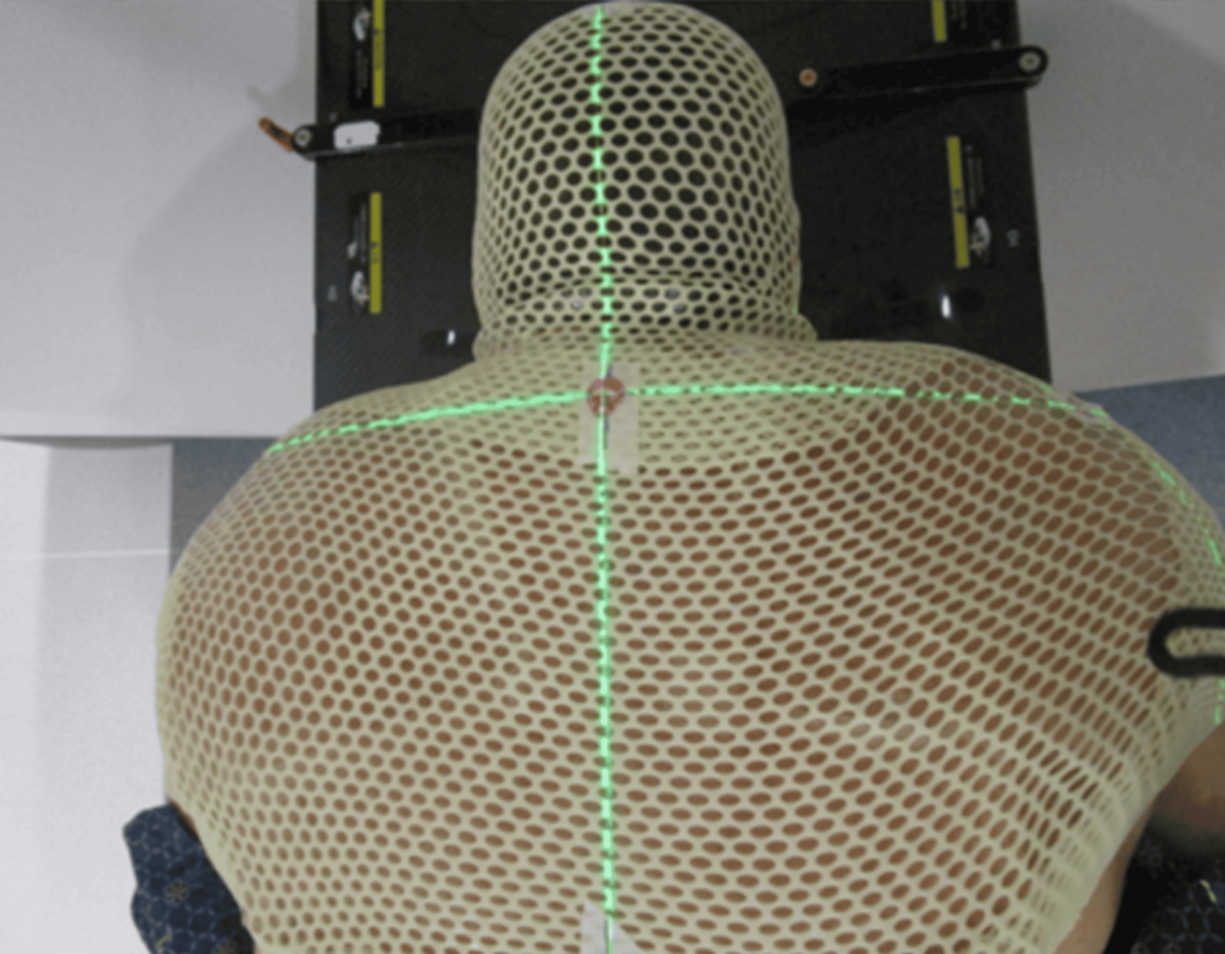
Treatment setup with mask This figure demonstrates the patient’s thermoplastic mask. The superflab bolus, partially visualized, is positioned under the mask, covering the neck and upper shoulder areas where the mask appears more opaque.

To address the dilemma of being unable to cover her areas of disease with an electron-based plan, photon-based volumetric modulated arc therapy (VMAT) was given over 20 Gy in 10 fractions for this seventh treatment, targeting the areas of active symptoms. CT imaging was used for planning, and a superflab bolus was applied to ensure adequate depth of coverage. Figures 1-4 and Figures 5-6 demonstrate treatment planning with VMAT and the treatment setup, respectively.

On the day of her second fraction of radiation, she experienced three episodes of generalized seizure-like activity in the context of hypoglycemia (blood glucose of 54). EEG revealed no epileptiform activity, and the CT head showed no concerning findings. By the sixth treatment, the exam already showed a supple neck with full ROM and no skin lesions remaining. She experienced no side effects from this photon treatment course.

Three months out from radiation, although her right upper extremity still felt heavy with occasional tingling of her hands and fingers along with continued bilateral shoulder pain at night (likely secondary to adhesive capsulitis), the patient was able to return to her work and reported that she no longer had impairment from her scleredema.

During a recent orthopedic visit, her right-sided shoulder demonstrated limited active and passive ROM: forward elevation of 120 degrees, external rotation of 20 degrees, and internal rotation of the abducted arm of 10 degrees. In comparison, her left side showed greater ROM: 130 degrees, 30 degrees, and 10 degrees, respectively. Her ROM limitations were determined to be mostly due to adhesive capsulitis; however, an MRI scan at that time also revealed a small anterior full-thickness supraspinatus tear without evidence of scleredema.

The pathophysiology underlying radiation’s effects on diabetic scleredema remains poorly understood; however, it is hypothesized that radiation may trigger anti-inflammatory effects, altering fibroblast production or fibroblast function in mucin and connective tissue production [7]. As exemplified in this case, despite an incomplete understanding of its mechanism of action, radiation therapy has demonstrated consistent short-term benefits in patients with diabetic scleredema compared to other available treatment modalities [7]. While limited literature supports any treatment modality for scleredema, phototherapy also stands out with several studies backing its use [3,6,10-15]. In contrast to the substantial but temporary improvements from radiation, this patient's scleredema was unresponsive to PUVA, low-dose methotrexate, and physical therapy. This case underscores radiation's superiority over other current modalities and suggests that scleredema does not develop resistance to radiation even after multiple courses. Moreover, while this patient had to discontinue low-dose methotrexate because of liver enzyme elevations and fatigue, only grade 1 skin toxicities of dermatitis, itching, and telangiectasia were experienced over her seven courses of radiation.

Although no established minimum necessary radiation dose exists, the literature suggests using a range of 18-24 Gy in 1.8 to 2 Gy fractions [7,18-20]. In the current case, the patient had a complete response to 12 Gy in six fractions, indicating that even low doses can have some effect on scleredema. However, given her relapse within less than a month with that low-dose treatment compared to several months of complete response with standard dosing, standard doses may be necessary for a sustained response. VMAT effectively treated the cylindrical surfaces of her shoulders and neck, and a bolus ensured dose buildup in the skin while sparing deeper tissues. Her positive results of VMAT-based therapy reveal the potential utility of VMAT for similar cases.

## Conclusions

While this study is limited to one isolated patient case and the findings may not be widely generalizable, it provides insights into optimal treatment strategies for scleredema diabeticorum. This case highlights the effectiveness of radiation compared to other modalities, the safety of re-irradiation for this disease, and the potential use of VMAT for anatomically challenging cases. In this case, the patient, who had no response to other treatment modalities, exhibited complete yet transient responses to multiple reradiation courses with minimal toxicity. The toxicity was limited to acute CTCAE grade 1 dermatitis and itching as well as delayed grade 1 telangiectasia. There was no treatment resistance even after the seventh course of radiation treatments.

This case supports the role of radiation as a safe, effective, and reliable treatment modality for patients with extensive and/or refractory disease. While there have been no documented cases in the literature of photon-only plans, VMAT made the patient's most recent seventh radiation course feasible and may be a viable option for other patients with disease distributions not amenable to electron radiation. The patient demonstrated no acute toxicity from this treatment and is currently over three months post-VMAT photon-based plan with no signs of disease or significant toxicity.
